# Subtelomeric I-SceI-Mediated Double-Strand Breaks Are Repaired by Homologous Recombination in *Trypanosoma cruzi*

**DOI:** 10.3389/fmicb.2016.02041

**Published:** 2016-12-22

**Authors:** Miguel A. Chiurillo, Roberto R. Moraes Barros, Renata T. Souza, Marjorie M. Marini, Cristiane R. Antonio, Danielle R. Cortez, María Á. Curto, Hernán A. Lorenzi, Alejandro G. Schijman, José L. Ramirez, José F. da Silveira

**Affiliations:** ^1^Laboratorio de Genética Molecular “Dr. Yunis-Turbay,” Decanato de Ciencias de la Salud, Universidad Centroccidental Lisandro AlvaradoBarquisimeto, Venezuela; ^2^Laboratory of Malaria and Vector Research, National Institute of Allergy and Infectious Diseases, National Institutes of Health, RockvilleMD, USA; ^3^Departamento de Microbiologia, Imunologia e Parasitologia, Escola Paulista de Medicina, Universidade Federal de São PauloSão Paulo, Brazil; ^4^Laboratorio de Biología Molecular de la Enfermedad de Chagas, Instituto de Investigaciones en Ingeniería Genética y Biología Molecular – Consejo Nacional de Investigaciones Científicas y TécnicasBuenos Aires, Argentina; ^5^Department of Infectious Diseases, J. Craig Venter Institute, RockvilleMD, USA; ^6^Centro de Biotecnología, Fundación Instituto de Estudios AvanzadosCaracas, Venezuela

**Keywords:** *T. cruzi*, telomere, I-SceI meganuclease, double-strand break, homologous recombination, DNA repair, artificial chromosomes

## Abstract

*Trypanosoma cruzi* chromosome ends are enriched in surface protein genes and pseudogenes (e.g., trans-sialidases) surrounded by repetitive sequences. It has been proposed that the extensive sequence variability among members of these protein families could play a role in parasite infectivity and evasion of host immune response. In previous reports we showed evidence suggesting that sequences located in these regions are subjected to recombination. To support this hypothesis we introduced a double-strand break (DSB) at a specific target site in a *T. cruzi* subtelomeric region cloned into an artificial chromosome (pTAC). This construct was used to transfect *T. cruzi* epimastigotes expressing the I-SceI meganuclease. Examination of the repaired sequences showed that DNA repair occurred only through homologous recombination (HR) with endogenous subtelomeric sequences. Our findings suggest that DSBs in subtelomeric repetitive sequences followed by HR between them may contribute to increased variability in *T. cruzi* multigene families.

## Introduction

*Trypanosoma cruzi* exhibits a vast repertoire of surface antigens encoded by ∼ 18% of all protein-coding genes, which are directly implicated in the parasite’s interaction with insect vectors and vertebrate hosts (El-Sayed et al., 2005; De Pablos and Osuna, 2012). The dramatic expansion and diversification of repetitive sequences, particularly of surface antigen gene families [trans-sialidase (TS), mucin, surface protein associated mucin (MASP), dispersed gene family-1 (DGF-1) and serine-, alanine- and proline-rich protein (SAP)], may have contributed to the speciation of *T. cruzi* (El-Sayed et al., 2005; Chiurillo et al., 2016).

*Trypanosoma cruzi* subtelomeric regions are enriched with genes and pseudogenes (ψ) of the TS, DGF-1 and retrotransposon hot spot (RHS) protein families (Moraes Barros et al., 2012), suggesting that these regions may act as sites for DNA recombination and the generation of new variants of surface protein genes without affecting the functional interstitial copies (Kim et al., 2005; Moraes Barros et al., 2012). Homologous chromosomes frequently show a loss of synteny in the subtelomeric region, reinforcing the hypothesis that recombination events could have arisen in *T. cruzi* subtelomeric regions (Moraes Barros et al., 2012).

Despite the high level of polymorphism in *T. cruzi* subtelomeric regions, an organizational pattern frequently observed in *T. cruzi* chromosome ends is the presence of TS genes and pseudogenes flanked by RHS sequences (Kim et al., 2005; Moraes Barros et al., 2012), resembling the organization of the repetitive regions adjacent to variant surface glycoprotein (VSG) genes in *T. brucei* telomeres (Hertz-Fowler et al., 2008). These (70-bp) repetitions are involved in recombination mechanisms responsible for antigenic variation in the African trypanosome (Boothroyd et al., 2009; Glover et al., 2013; Li, 2015).

Although recombination-mediated VSG switching is rare in recently laboratory-adapted parasite lines, the induction of a double-strand break (DSB) adjacent to the 70-bp repetitions of the telomeric VSG locus by I-SceI meganuclease leads to an up to 250-fold increase in the exchange of these genes (Boothroyd et al., 2009). Spontaneous DSBs in 70-bp repetitive regions, particularly in the active expression site (ES), can promote DNA recombination-mediated VSG switching involved in *T. brucei* antigenic variability (Glover et al., 2013; Li, 2015). The location of the DSB in the ES determines the type of VSG switching mechanism that will take place (Glover et al., 2013). Although the nature of subtelomeric repetitive sequences in *T. brucei* and *T. cruzi* is different, a similar mechanism may exist in *T. cruzi*, leading to the surface antigenic variability found in this parasite.

Recently, a linear vector series termed pTAC, for Trypanosoma Artificial Chromosome, has proven to be a useful set of tools for genetic studies in *T. cruzi* because of the high stability of these vectors as linear episomes and their faithful segregation in all the parasite developmental forms (Curto et al., 2014). In the study by Curto et al. (2014) *T. cruzi* epimastigotes were transfected with a pTAC carrying a 1.7-kb fragment of a subtelomeric ψTS sequence, and after transgenic parasites were subjected to different conditions, no recombination events involving the displacement of the ψTS sequence from the pTAC to the parasite genome were detected. Their results therefore suggest that the presence of a ψTS sequence in the construct was not sufficient to induce recombination between the artificial chromosome pTAC and an endogenous chromosome. The absence of these DNA rearrangement events involving the ψTS sequence suggests that other elements of the subtelomeric region or additional chromosomal events may be necessary to promote recombination processes.

In the present study we aimed to determine whether a DSB in subtelomeric repetitive sequences induces recombination involving the subtelomeric regions using a two-step approach: generation of *T. cruzi* cell lines expressing the I-SceI meganuclease and subsequent transfection of these cells with a stable linear vector (pTAC) carrying a *T. cruzi* subtelomeric region with the I-SceI recognition site.

## Materials and Methods

### Vector Construction

#### Linear Transgene Constructs (pTACs) Carrying the Recognition Site for I-SceI Meganuclease in the Subtelomeric Sequence

**FIGURE 1:**
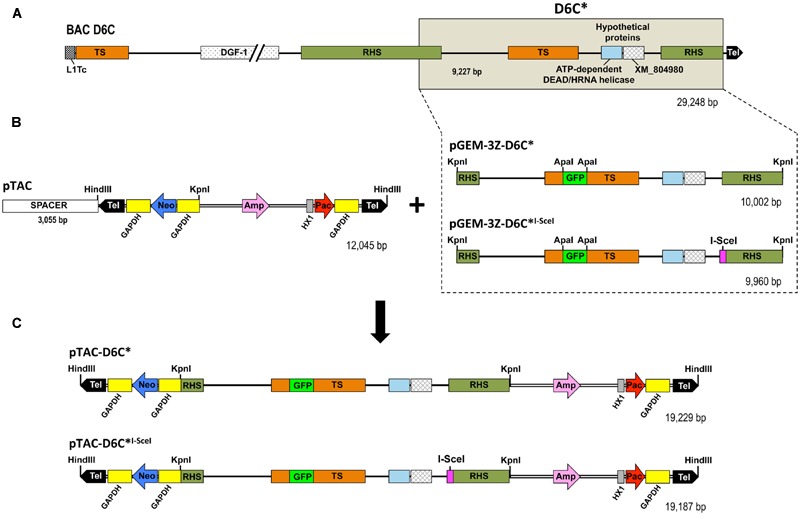
**Schematic representation of sequences and vectors used to generate pTAC constructs harboring a *T. cruzi* subtelomeric region. (A)** BAC-D6C, *T. cruzi* telomeric region (∼29 kb) cloned into pBeloBAC11 (Kim et al., 2005). **(B)** The shaded box in BAC-D6C (D6C^∗^ region, nt positions 19166 to 28426) represents the subcloned region in pGEM-3Z (pGEM-3Z-D6C). An artificial I-SceI meganuclease recognition site was introduced adjacent to the 5’ end (magenta square) of the RHS pseudogene (D6C^∗I-SceI^) by overlap extension PCR, resulting in a fragment 42 nucleotides smaller (10,002 *vs.* 9,960 bp). The GFP gene (from pEGFP plasmid; Clontech, USA) was inserted into the ApaI restriction site in the TS pseudogene to trace recombination events. D6C^∗^ and D6C^∗I-SceI^ fragments were cloned into the pTAC vector (10 kb), which has a unique KpnI cloning site for a DNA fragment of interest, keeping the neomycin (Neo) and puromycin (Pac) resistance genes in both arms. When circular plasmid is digested with HindIII restriction enzyme an ∼3-kb spacer fragment is excised and the resulting linearized vector is flanked by inverted *T. cruzi* telomeric and subtelomeric sequences (Tel: 900 bp each). **(C)** pTAC vectors harboring D6C^∗^ or D6C^∗I-SceI^ regions (19,229/19,187 bp, respectively) inserted at the KpnI restriction site. Relevant elements: L1Tc, *T. cruzi* non-long terminal repeat retrotransposon; TS, trans-sialidase; DGF-1, dispersed gene family-1; RHS, retrotransposon hot spot; GFP, green fluorescent protein; Amp, ampicillin; HX1, intergenic region of the TcP2ß gene; GAPDH, intergenic region of glyceraldehyde-3-phosphate dehydrogenase.

The design of the constructs for this purpose is shown in **Figure 1**. An ∼9 kb *T. cruzi* subtelomeric fragment cloned into the recombinant bacterial artificial chromosome BAC-D6C (Kim et al., 2005; GenBank accession number AY551440) was subcloned into the plasmid pGEM-3Z. The insert, called D6C^∗^, included a TS pseudogene flanked by truncated copies of RHS. An I-SceI recognition site was added upstream of the second RHS copy by an overlap extension PCR strategy that generated the D6C^∗I-SceI^ DNA fragment. The resulting fragments D6C^∗^ and D6C^∗I-SceI^ were inserted into the KpnI cloning site of pTAC to obtain the pTAC-D6C^∗^ and pTAC-D6C^∗I-SceI^ constructs.

#### Integrative *T. cruzi* Expression Vector (pTREX) Encoding the I-SceI Meganuclease Fused to a Nuclear Localization Signal (NLS)

The open reading frame (ORF) of I-SceI meganuclease was subcloned in fusion with the GFP (green fluorescent protein) gene of the vector pTREXn-GFP5(S65T; Vazquez and Levin, 1999; Guevara et al., 2005). To import the I-SceI protein into the parasite nucleus, the nuclear localization signal (NLS) from SV40 large T antigen or *T. cruzi* histone H2B (TcH2B; Marchetti et al., 2000) was fused to the N-terminus of the I-SceI protein. (SV40)I-SceI ORF was PCR amplified from the pLew100-NLS-ISceI-HA vector (kindly provided by Dr Nina Papavasiliou, Rockefeller University) while the (TcH2B)I-SceI fusion gene was synthesized by GenScript (USA). To determine the cellular localization of I-SceI meganuclease we made a construct placing the GFP gene reporter downstream of the I-SceI meganuclease ORF.

#### Parasite Culture and Transfection

Epimastigotes of *T. cruzi* clone CL Brener (CLB) were maintained in axenic cultures at 28°C in liver-infusion tryptose (LIT) medium supplemented with 10% heat-inactivated fetal calf serum (Camargo, 1964). They were transfected with pTREX-GFP, pTREX-(SV40)I-SceI-GFP or pTREX-(TcH2B)I-SceI-GFP plasmids following Vazquez and Levin (1999). Transfectants were selected in the presence of G418 at 500 μg/mL in LIT medium with 10% fetal bovine serum. Next, epimastigotes expressing I-SceI meganuclease or not were transfected with pTAC, pTAC-D6C^∗^ or pTAC-D6C^∗I-SceI^ vectors and grown in the presence of G418 (100 μg/mL) and puromycin (10 μg/mL; Curto et al., 2014). Double transfectants were then cloned by serial dilution in a 96-well plate, and clones were evaluated by fluorescence microscopy to confirm expression of GFP in the nucleus of the mutant cell lines.

#### I-SceI Meganuclease Activity

Nuclear extracts of epimastigotes were obtained according to the method described by Vergnes et al. (2007) with modifications. Briefly, 10^10^ epimastigotes were lysed and after centrifugation at 3000 × *g* the pellet was resuspended in a nuclear extraction buffer (20 mM HEPES pH 7.5; 0.4 M NaCl; 1.5 mM MgCl_2_; 0.2 mM EDTA; 25% glycerol; 1 mM PMSF; 1 mM DTT), kept on ice for 30 min and centrifuged at 12,000 × *g* for 30 min at 4°C. The supernatant was recovered, and protein concentration was estimated by the Bradford Protein Assay (Bio-Rad). To determine the presence of I-SceI meganuclease activity in the transfected epimastigotes, a DNA fragment containing an I-SceI restriction site was incubated with the nuclear extract at 37°C overnight, and the digested DNA samples were subsequently analyzed by agarose gel electrophoresis.

#### PCR Analysis of *T. cruzi* Mutant Cell Lines

Epimastigote pellets (10^8^ cells) were resuspended in lysis buffer (150 mM NaCl; 250 mM EDTA; 100 μg/mL proteinase K; 0.5% sarkosyl; 1 MTris-HCl, pH 8.0), followed by DNA extraction with phenol:chloroform and treatment with RNAse A (100 μg/mL). DNA amplification was carried out using Platinum Taq DNA Polymerase High Fidelity (Invitrogen). Two primer sets were used to detect the repair events: F1 (5′-TTTCCCTGTGAGAGCTTACC-3′)/R1 (5′-ACTATAGGGCGAATTGGG-3′) and F2 (5′-CAGAGTGTGTGCGGTGTC-3′)/R2 (5′-CGTCCATGACTGCGGTGG-3′). Amplicons produced by F1/R1 were purified using an agarose gel extraction kit, cloned into the pGEM-T easy vector system (Promega) and subsequently subjected to DNA sequencing using primers F2/R2.

#### Pulsed-Field Gel Electrophoresis (PFGE) and Southern Blot Analysis

*Trypanosoma cruzi* chromosomal DNA was resolved by pulsed-field gel electrophoresis (PFGE) using a Gene Navigator System (Pharmacia) with a hexagonal electrode array as previously reported (Cano et al., 1995). Chromosomal preparations of *T. cruzi* transgenic cell lines containing pTAC constructs were separated by clamped homogeneous electric field (CHEF) gel electrophoresis using a CHEF Mapper^®^ XA System (Bio-Rad) on 1% DNA typing-grade agarose (Gibco BRL) gels and 0.5X TBE buffer. Electrophoresis was carried out for 18 h at 14°C with an electrode angle of 120°, a constant voltage of 6 V/cm and a switch time of 60 to 90 s. For the restriction fragments of epimastigote genomic DNA digested with the HindIII and KpnI, the same conditions were used but with a switch time of 1 to 6 s and run time of 12 h. After electrophoresis, DNA was stained with ethidium bromide (0.5 μg/mL), transferred to nylon filters and hybridized with ^32^P-labeled probes as previously described (Martins et al., 2015).

## Results

**FIGURE 2:**
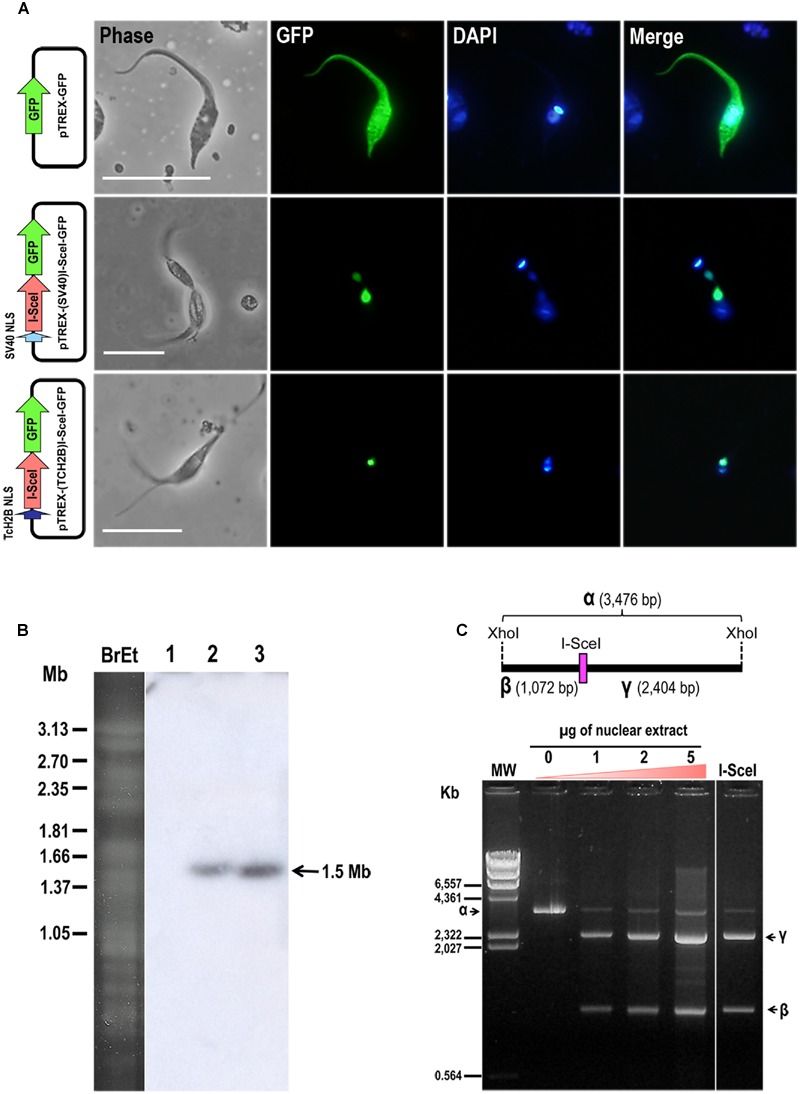
***T. cruzi* epimastigotes expressing I-SceI meganuclease enzyme. (A)** Fluorescence microscopy images of *T. cruzi* (clone CL Brener) epimastigotes transfected with pTREX-GFP, pTREX-(SV40)I-SceI-GFP or pTREX-(TcH2B)I-SceI-GFP constructs as indicated on the left side. From left to right side are shown images from phase-contrast, GFP fluorescence (green), DAPI (blue) and merged between GFP and DAPI. Bars = 10 μm. **(B)** Electrokaryotype of *T. cruzi* cell lines harboring pTREX-I-Sce-I constructs. Chromosomal bands were separated by PFGE and hybridized with a ^32^P-radiolabeled I-SceI gene (700 bp) probe. BrEt, agarose gel stained with ethidium bromide; (1) wild-type epimastigotes; (2) epimastigotes transfected with pTREX-(SV40)I-SceI-GFP; and (3) pTREX-(TcH2B)I-SceI-GFP. The arrow on the right indicates an ∼1.5-Mb hybridizing chromosomal band. **(C)** I-SceI activity in nuclear extracts of *T. cruzi* epimastigotes. A schematic representation is shown above the picture of the agarose gel. The I-SceI restriction site in a XhoI-XhoI 3,476-bp DNA fragment(α) is indicated. The fragment was incubated overnight at 37°C with increasing amounts of nuclear extract obtained from epimastigotes harboring pTREX-(SV40)I-SceI-GFP construct. This digestion resulted in two restriction fragments (β and γ), which were separated by electrophoresis in a 1% agarose gel. As a control, fragment α was also digested with commercial I-SceI enzyme (Fermentas), and the resulting fragments were loaded into the last lane of the gel. The arrows indicate migration of fragments α, β and γ on the gel. MW, molecular weight marker.

We successfully inserted a single I-SceI restriction site in a *T. cruzi* subtelomeric region that was then subcloned into the pTAC plasmid to generate the construct pTAC-D6C^∗I-SceI^ (**Figure 1**). We also used the vector pTREXn-GFP5(S65T; Vazquez and Levin, 1999; Guevara et al., 2005) and either SV40 or TcH2B NLSs to constitutively express I-SceI meganuclease in *T. cruzi.* Epimastigotes transfected with SV40- or TcH2B-I-SceI-GFP allowed us to demonstrate that the I-SceI meganuclease, as expected, had accumulated in the parasite’s nucleus (**Figure 2A**). Chromosomal bands of epimastigotes transfected with pTREX-(SV40)I-SceI-GFP or pTREX-(TcH2B)I-SceI-GFP were separated by PFGE and hybridized with an I-SceI gene probe (**Figure 2B**). A hybridization signal with an approximately 1.5-Mb band was observed in both mutant cell lines, showing that the nuclease gene had been integrated into the *T. cruzi* nuclear genome and confirming the integrative nature of pTREX, as previously described by Lorenzi et al. (2003). Since the nuclease was efficiently imported into the parasite nucleus by both NLSs, we chose to continue the work using the pTREX-(SV40)I-SceI-GFP construct. We further confirmed the activity of I-SceI meganuclease expressed in *T. cruzi* by incubating a DNA fragment carrying an I-SceI restriction site with the nuclear extract of epimastigotes and then separating the digested DNA by agarose gel electrophoresis (**Figure 2C**).

After selection, we carried out a second round of transfection in wild type cells or in a homogeneous fluorescent cell population transfected with pTREX-(SV40)I-SceI-GFP construct. Epimastigotes expressing I-SceI meganuclease (homogeneous population exhibiting green fluorescent nuclei) or not were transfected with the vectors pTAC, pTAC-D6C^∗^ or pTAC-D6C^∗I-SceI^ and grown in the presence of G418 (100 μg/mL) and puromycin (10 μg/mL). Double transfectants were then cloned by serial dilution in a 96-well plate and evaluated by fluorescence microscopy to confirm green fluorescence in their nuclei. The transfected parasites, expressing I-SceI meganuclease or not, were further analyzed by PFGE followed by Southern blot using a GFP probe that tagged the TS pseudogene in both constructs. Using chromoblot hybridization we confirmed the presence of pTAC molecules as episomal copies with no apparent recombination with the genomic DNA (**Supplementary Figure S1A**). Furthermore, analysis of total genomic DNA digested with HindIII or KpnI enzymes showed that restriction fragments of artificial chromosomes had the expected original size (**Supplementary Figure S1B**).

**FIGURE 3:**
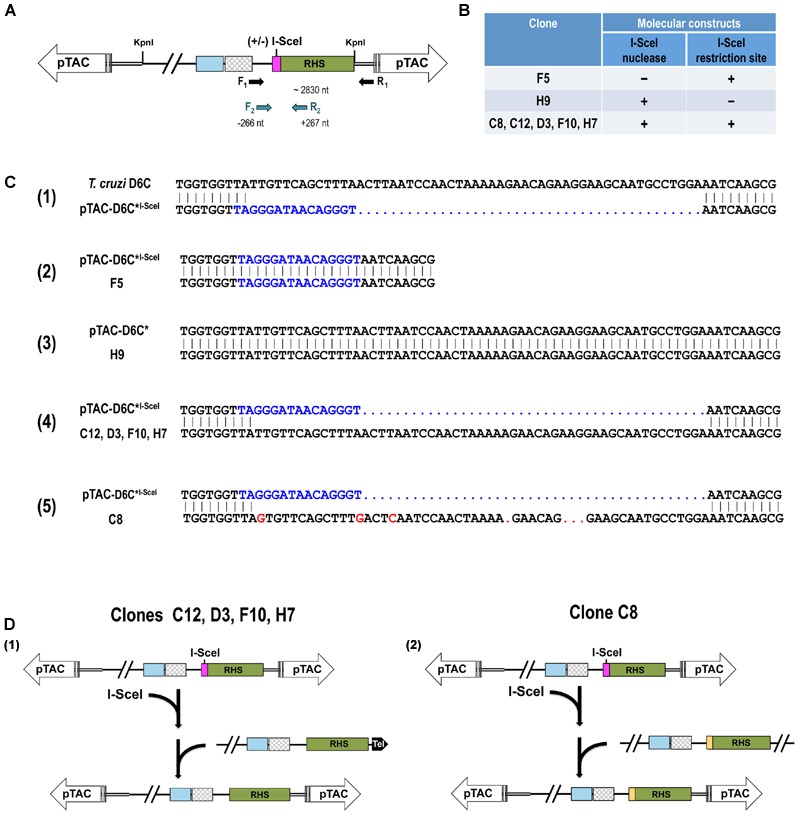
**Analysis of double-strand break repair events. (A)** Schematic representation of PCR strategy used to amplify a 2.8-kb fragment of pTAC-D6C^∗I-SceI^ sequence spanning the I-SceI site in mutant *T. cruzi* epimastigotes. Fragments amplified with primers F1 and R1 were cloned into pGEM-T, and a nested PCR based on primers F2 and R2 was used to sequence the DSB region. **(B)** Summary table showing molecular constructs present in cloned cell lines of *T. cruzi* transfected-epimastigotes. **(C)** Nucleotide sequence alignment of the region adjacent to the DSB site in the wild-type *T. cruzi* telomeric sequence cloned into BAC-D6C with the D6C^∗I-SceI^ sequence including the I-SceI restriction site (in blue) **(1)**; pTAC-D6C^∗I-SceI^ vector with pTAC-D6C^∗I-SceI^ mutant clone F5 **(2)**; pTAC-D6C^∗^ vector with pTREX-(SV40)I-SceI-GFP/pTAC-D6C^∗^ mutant clone H9 **(3)**; pTAC-D6C^∗I-SceI^ vector with pTREX-(SV40)I-SceI-GFP/pTAC-D6C^∗I-SceI^ mutant clones C12, D3, F10 and H7 **(4);** pTAC-D6C^∗I-SceI^ vector with pTREX-(SV40)I-SceI-GFP/pTAC-D6C^∗I-SceI^ mutant clone C8 **(5)**. **(D)** Representation of DSB repair events by homologous recombination (HR). Recombination between regions flanking the I-SceI site in the pTAC-D6C^∗I-SceI^ molecule may have led to loss of the I-SceI site and replacement of this site with genomic sequences. Schemas **(1,2)** are representations of events shown in **(B)**.

Next, we randomly selected seven transgenic parasite clones for further analysis, five of which expressed I-SceI enzymatic activity and carried the I-SceI recognition site (pTREX-(SV40)I-SceI-GFP/pTAC-D6C^∗I-SceI^). Of the remaining mutants, one did not have I-SceI enzymatic activity because it was only transfected with pTAC-D6C^∗I-SceI^, and the other expressed I-SceI enzymatic activity but lacked the recognition site for I-SceI (pTREX-(SV40)I-SceI-GFP/pTAC-D6C^∗^). The region containing the I-SceI recognition site was sequenced to assess whether it was involved in any DSB repair events (**Figure 3**). To avoid genomic DNA amplification, repair events were identified by PCR amplification in total parasite DNA using forward primer F1, which anneals upstream of the I-SceI nuclease site, and reverse primer R1, which anneals to the pTAC sequence. The target/DSB site in the amplicons was sequenced by nested PCR using a second forward primer (F2) that anneals to a few nucleotides internal to F1 and a reverse primer (R2) that anneals within the RHS gene (**Figure 3A**).

**Figure 3B** summarizes the presence of the I-SceI gene and I-SceI meganuclease recognition site in the seven selected clones from transfected cell lines. Sequences derived from epimastigotes that carry the pTAC-D6C^∗I-SceI^ vector but do not express the heterologous I-SceI meganuclease and from those expressing the enzyme but transfected with pTAC-D6C^∗^ clearly displayed the unmodified sequence used for each transfection (**Figures 3B** and **3C-2,3**). In all parasite clones analyzed in this work that express the nuclease and harbor the I-SceI recognition site, we observed that the DSBs were repaired, probably by homologous recombination (HR) mechanism. The absence of nucleotide insertions or deletions in the repaired sequences allowed us to rule out the occurrence of non-homologous end-joining (NHEJ) or microhomology-mediated end-joining (MMEJ) events (**Figures 3B** and **3C-4,5**). We found that all DSBs were repaired, and that in 80% (4/5) of junctions the non-mutated parental endogenous sequence, which is identical to the wild-type (D6C) sequence (**Figure 3C-4**), was involved in this repair. However, clone C8 sequence showed the involvement of a donor with sequence similarity to the non-mutated parental sequence (88.1% of identity in the repaired region; **Figure 3C-5**). Interestingly, the latter sequence displayed >98% of identity with a subtelomeric sequence of *T. cruzi* CLB (tritryp.org, genomic sequence ID: TcChr11-S). In this case, the recombination event occurred in a region comprising the 3′ end of an RHS gene (TcCLB.509349.30 – locus 115,973…117,865 nt) and the intergenic region between the RHS gene and a gene encoding a hypothetical protein (TcCLB.509349.20 – locus 118,491…119012). This region is 117,849 nt from the telomeric repeat located in the subtelomeric region of Tel 2 (TcChr11S; **Supplementary Figure S2**) described by Moraes Barros et al. (2012). This result lends support to the hypothesis of the existence of recombination mechanisms between subtelomeric regions of the parasite that could generate variations in surface protein genes.

**Figure 3D** shows the HR events that may have occurred to repair the DSBs induced in the pTAC-D6C^∗I-SceI^ vector. Recombination between I-SceI-site-flanking regions in pTAC-D6C^∗I-SceI^ and the parasite genome may have led to loss of the I-SceI site and its replacement with genomic sequences. In our experiments the most common recombination event occurred between the artificial chromosome pTAC and the *T. cruzi* chromosome end from which the telomere cloned in the BAC-D6C was derived (**Figures 3C-4** and **3D-1**). However, mutant clone C8 was generated by an ectopic HR event with a non-parental subtelomeric sequence (**Figures 3C-5** and **3D-2**).

## Discussion

Repair of DSBs in lower eukaryotes, such as trypanosomatids, occurs preferentially by HR in the presence of an appropriate DNA donor with homologous sequences (Bhattacharyya et al., 2004; Passos-Silva et al., 2010; Glover et al., 2011). In fact, key elements of HR repair mechanisms have been identified in the genome of these trypanosomatids (El-Sayed et al., 2005; Passos-Silva et al., 2010). Furthermore, in procyclic forms of *T. brucei* a minimal 42 bp of homology has been observed to be sufficient for HR-mediated DNA integration (Gaud et al., 1997).

NHEJ is absent in trypanosomatids (Passos-Silva et al., 2010; Glover et al., 2011), and only a few factors involved in this type of DNA repair mechanism can be identified in these organisms. Moreover, MMEJ has also been identified in *T. brucei* and *T. cruzi*, although in the latter the mechanism has not been fully elucidated. MMEJ is usually considered a backup DSB repair pathway in the absence of a DNA template, although HR can be more frequent and efficient than MMEJ (Conway et al., 2002; Barnes and McCulloch, 2007; Glover et al., 2008, 2011; Passos-Silva et al., 2010; Peng et al., 2014; Lander et al., 2015). MMEJ can be distinguished from NHEJ by the fact that it uses 3 to 20 bp of microhomology to repair DSBs, leaving deletions of variable sizes between the microhomology regions (Conway et al., 2002; Peng et al., 2014). Similarly, a recent study has shown that *Plasmodium falciparum* uses two mechanisms to repair DNA DSBs (Kirkman et al., 2014). In the first, when a suitable donor molecule is available, chromosomal DSBs are exclusively repaired by HR. In contrast, when no homologous sequence is available, DSBs are repaired by a non-canonical NHEJ pathway, which has features similar to synthesis-dependent microhomology-mediated repair mechanisms (Yu and McVey, 2010).

In human trypanosomes (*T. brucei* and *T. cruzi*), HR plays an important role in rearrangements and changes in the genome that form the basis of useful strategies for survival in the host (Conway et al., 2002; Westenberger et al., 2005; Baptista et al., 2014). The switch of one VSG in the ES in *T. brucei* telomeres can be determined by telomere exchange/crossover or gene conversion mechanisms. Therefore, HR plays a role in the antigenic variation undergone by this parasite to evade the vertebrate host immune response (Li, 2015). In addition, spontaneous DSBs can activate VSG switching by HR-mediated DSB repair mechanisms (Boothroyd et al., 2009). Similarly, it has been suggested that HR events contribute to the high variability in genes encoding for surface proteins (e.g., mosaic genes) in *T. cruzi*, thus increasing the stock of molecules that are involved in evasion of the host immune system, participate in cell invasion and contribute to virulence (Machado et al., 2006; Cerqueira et al., 2008).

In this work we employed pTAC vectors as a tool to promote and analyze the exchange of genetic material focusing on the subtelomeric region of *T. cruzi*. As previously demonstrated, pTAC vectors can exist as highly stable linear extrachromosomal elements, showing accurate replication and segregation comparable to that of natural *T. cruzi* chromosomes (Curto et al., 2014). We were also able to introduce a DSB at a targeted subtelomeric locus carried in an artificial chromosome by means of heterologous expression of yeast meganuclease I-SceI even though *T. cruzi* is generally considered to be refractory to genetic manipulation. Sequence analysis indicated that DSBs in the examined clones were repaired exclusively by an HR mechanism using homologous genomic subtelomeric regions as DNA templates. Although the ψTS and RHS sequences flanking the I-SceI site remained intact, our preliminary results with a small number of clones provide evidence to suggest that DSBs in subtelomeric regions, which are considered fragile and prone to breaks, are repaired by a HR mediated mechanism. Therefore, DSBs may play a role in generating variability in repetitive sequences and in telomere polymorphism in *T. cruzi*. It would be interesting to test the generation of DSBs in other sites along the subtelomeric region. In this regard, by facilitating insertion of restriction sites into endogenous genomic sequences and other sequence elements, the recent implementation of CRISPR/Cas9 technology in *T. cruzi* (Peng et al., 2014; Lander et al., 2015) can soon be expected to benefit studies of the role of subtelomeric regions in mechanisms operating in *T. cruzi* that generate DNA exchange.

## Author Contributions

Conceived and designed experiments: MC, RM, RS, and JdS. Performed the experiments: MC, RM, RS, MM, CA, DC, MC, and HL. Analyzed or interpreted the data: MC, AS, JR, and JdS. Wrote the paper: MC and JdS wrote the paper with contributions of all authors. All authors read and approved the final manuscript.

## Conflict of Interest Statement

The authors declare that the research was conducted in the absence of any commercial or financial relationships that could be construed as a potential conflict of interest.

## Supplementary Material

FIGURE S1 **Characterization of pTAC constructs in transfected epimastigotes.** In order to follow episomal pTAC DNA rearrangements in, or insertion into, the *T. cruzi* genome, epimastigote chromosomal preparations **(A)** and restriction fragments of genomic DNA digested with HindIII or KpnI **(B)** were separated by clamped homogeneous electric field (CHEF) gel electrophoresis using a CHEF Mapper^®^ XA System (Bio-Rad) under the conditions specified in the Matherial and Methods section and hybridized with a ^32^P-radiolabeled GFP probe corresponding to the sequence used to tag the TS pseudogene in the D6C^∗^/ D6C^∗I-SceI^ sequence. *T. cruzi* cell lines included in both analyses: (1) wt; (2) pTAC; (3) pTAC-D6C^∗^; (4) pTAC-D6C^∗I-SceI^; (5) pTREX-(SV40)I-SceI-GFP/pTAC-D6C^∗^; (6) pTREX-(SV40)I-SceI-GFP/pTAC-D6C^∗I-SceI^. The hybridization signals observed in **(B)** correspond to the restriction fragments expected for pTAC-D6C^∗^ and pTAC-D6C^∗I-SceI^ digestion with HindIII and KpnI (∼19 and 10 kb, respectively), as shown in the map of the pTAC-D6C^∗I-SceI^ construct.

FIGURE S2 **Nucleotide sequence alignment of clone C8 sequence including the repaired DSB region (in blue) with the subtelomeric TcChr11-S sequence of *T. cruzi* CL Brener**.
