# Supplementary figures and images for: Subtelomeric I-SceI-Mediated Double-Strand Breaks Are Repaired by Homologous Recombination in Trypanosoma cruzi
Source: Front Microbiol. 2016 Dec 22;7:2041. doi: 10.3389/fmicb.2016.02041 (PMC5177640; doi:10.3389/fmicb.2016.02041)

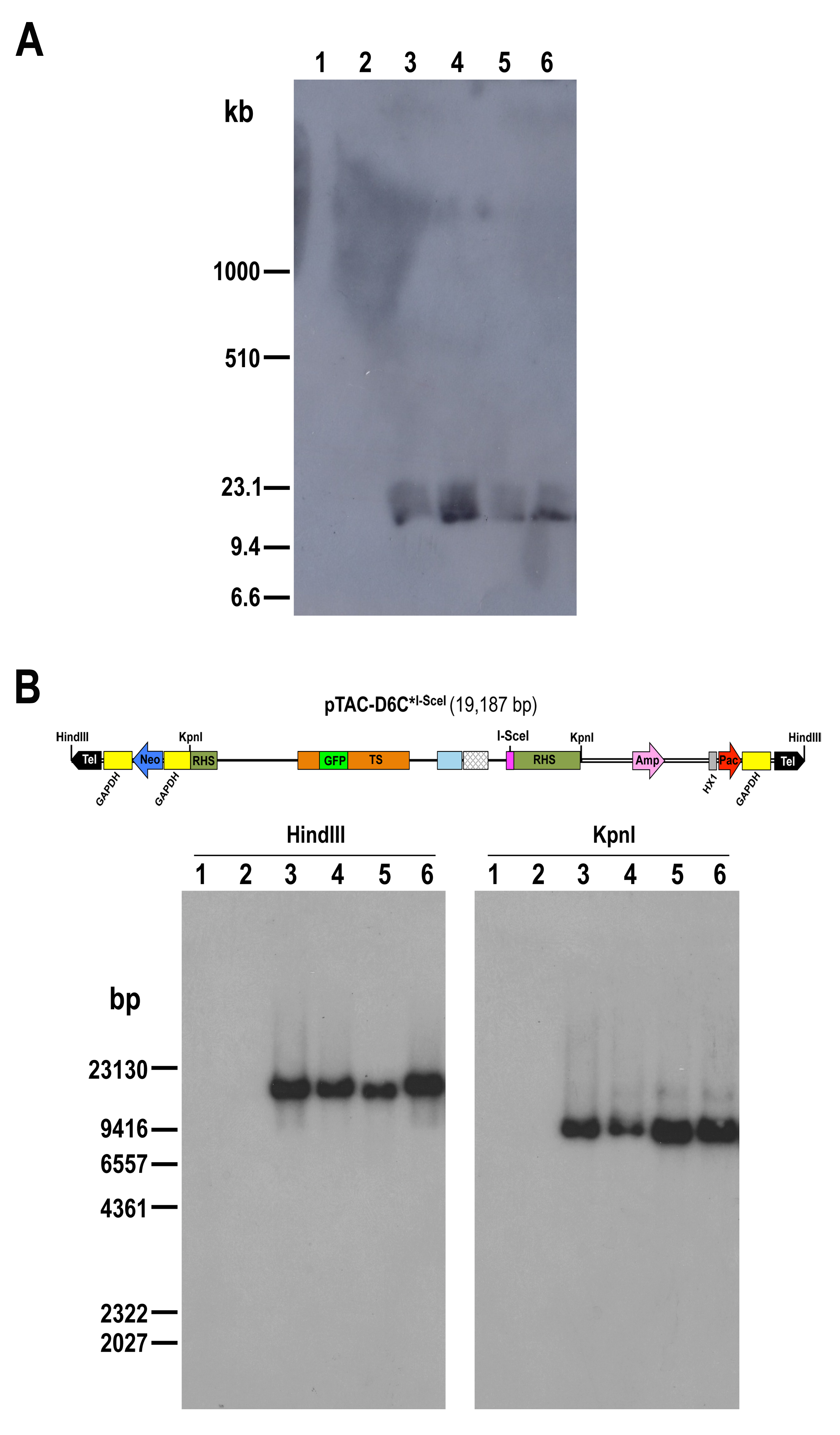

Supplement: FIGURE S1 — Characterization of pTAC constructs in transfected epimastigotes. In order to follow episomal pTAC DNA rearrangements in, or insertion into, the T. cruzi genome, epimastigote chromosomal preparations (A) and restriction fragments of genomic DNA digested with HindIII or KpnI (B) were separated by clamped homogeneous electric field (CHEF) gel electrophoresis using a CHEF Mapper® XA System (Bio-Rad) under the conditions specified in the Matherial and Methods section and hybridized with a 32P-radiolabeled GFP probe corresponding to the sequence used to tag the TS pseudogene in the D6C∗/ D6C∗I-SceI sequence. T. cruzi cell lines included in both analyses: (1) wt; (2) pTAC; (3) pTAC-D6C∗; (4) pTAC-D6C∗I-SceI; (5) pTREX-(SV40)I-SceI-GFP/pTAC-D6C∗; (6) pTREX-(SV40)I-SceI-GFP/pTAC-D6C∗I-SceI. The hybridization signals observed in (B) correspond to the restriction fragments expected for pTAC-D6C∗ and pTAC-D6C∗I-SceI digestion with HindIII and KpnI (∼19 and 10 kb, respectively), as shown in the map of the pTAC-D6C∗I-SceI construct. [file Image_1.TIF]

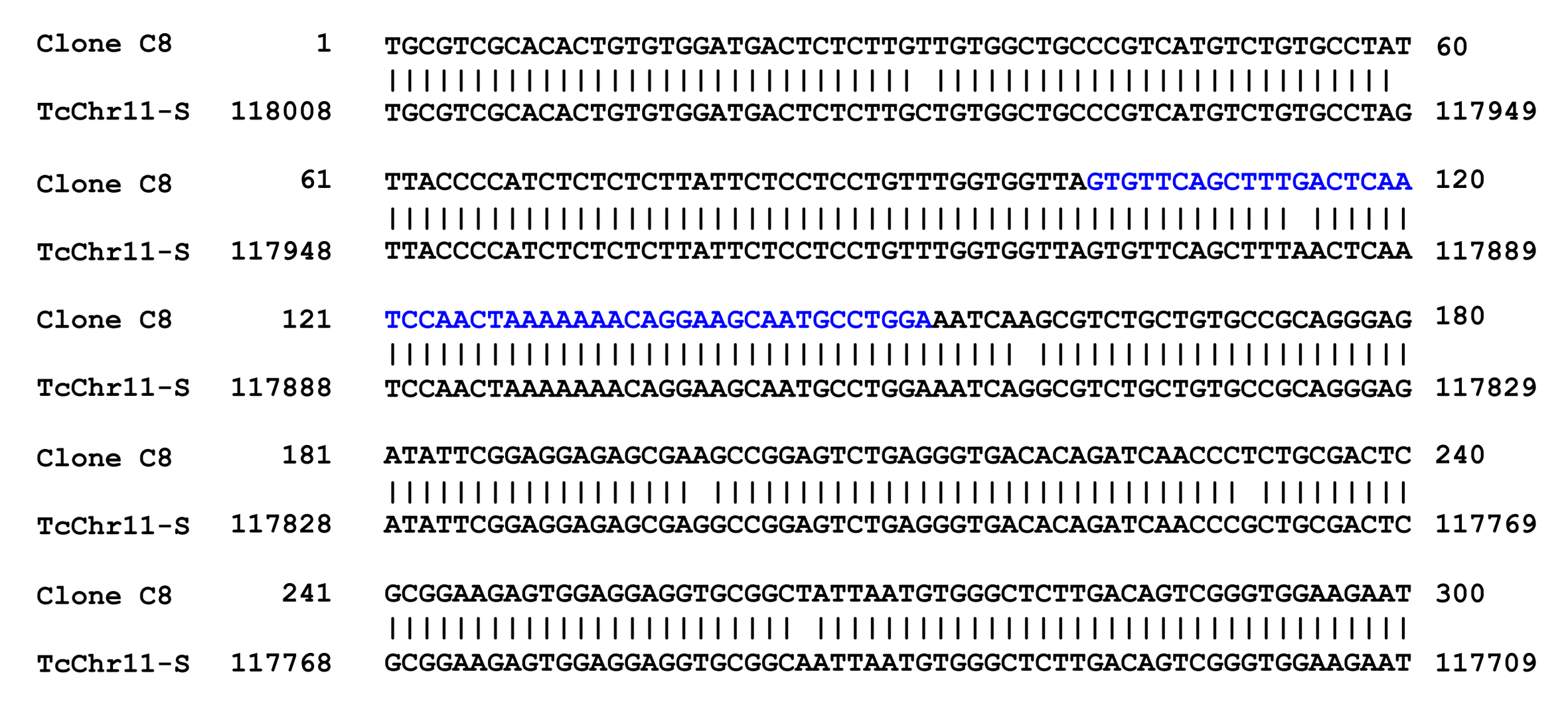

Supplement: FIGURE S2 — Nucleotide sequence alignment of clone C8 sequence including the repaired DSB region (in blue) with the subtelomeric TcChr11-S sequence of T. cruzi CL Brener. [file Image_2.TIF]
